# O17 Physiotherapy experience of treating patients with PIMS-TR COVID-19 following stepdown from PICU

**DOI:** 10.1093/rap/rkaa054.005

**Published:** 2020-11-03

**Authors:** Susan Maillard, Kim Noar, Samantha Harris, Jeremy Hasson, Kathryn Foy

**Affiliations:** Great Ormond Street Hospital NHS Foundation Trust, London, United Kingdom

## Abstract

**Case report - Introduction:**

It has been reported by the European Centre for Disease Prevention and Control that by May 2020 there were 230 suspected cases of PIMS-TR COVID-19. At Great Ormond Street Hospital NHS Foundation Trust (GOSH) there were 50 suspected cases. This report summarises the physiotherapy input to these patients outside of PICU. The rheumatology team were allocated the COVID-19positive ward and therefore were able to be involved in the evolving understanding of the clinical presentation and management of this new disease.

**Case report - Case description:**

50 children were admitted to GOSH with suspected PIMS-TR COVID-19, of these, 36 were admitted to PICU. The mean length of stay in PICU was 4.6 days and the median 3.5 days (Range 1–16 days).

The mean total length of stay in hospital was 11 days (range 2–94 days).

42% were male and 57% female and the age ranged from 5 weeks -17 years, but the mean age was 9 yrs. Most were previously well, but 3 had asthma, 2 diabetes, 1 obese, 1 with liver disease and 2 with sickle cell disease.

The physiotherapy provided included assessment and relatively quickly it was recognised that these patients had a specific pattern of muscle weakness (proximal>distal) and so the Manual Muscle Test of 8 muscle groups (MMT8) and the Childhood Myositis Assessment Score (CMAS) were adopted as outcomes. The initial mean MMT8 score was 56/80 (42–79) and mean CMAS score 20/52 (4–51)

Assessment of respiratory function, mobility and safety of postural changes including sitting to standing, gait and managing the stairs was also included.

The treatment provided included breathing exercises and specific muscle strengthening that was progressed as able. Advice to the nursing and medical staff was provided to ensure that patients were safe while they were regaining strength and stability. Gait re-education and stairs assessment was completed before discharge.

The patients have now been placed into a multidisciplinary assessment programme to follow up the long-term outcomes including physiotherapy outcomes. At the 2 months follow up the mean MMT8 was 72/80 and the mean CMAS is 46/52 indicating that there may be a long-term impact upon musculoskeletal function in young people.

**Case report - Discussion:**

At GOSH the rheumatology physiotherapy team were redeployed to the temporary general paediatrics service. This service was responsible for the patients who were diagnosed with this new and evolving disease and who were transferred from the PICU. The physiotherapy team started to recognise the extent of their illness including postural instability, muscle weakness, severe fatigue, and joint involvement. The children also had impairment in respiratory function and cardiac function. It was recognised that mobility was limited for many reasons and care was required in the intensity and frequency of exercise and level of activity. Initially moving around the bed was exhausting and had to be effective and safe before progressing to weight-bearing and walking.

Because the physiotherapy team were musculoskeletal specialists, they were able to consider different outcome measures and quickly decided upon using the MMT8 and the CMAS as well as assessing joint range of movement and muscle length. Respiratory assessments were also completed.

It was also recognised that as the hospital had rapidly developed the COVID-19ward (Hedgehog ward) and as the staff were from many different areas of the hospital effective communication between this new team had to be established and within weeks a daily MDT meeting was started that ensured all aspects of each patients care were discussed to enable complete co-ordinated treatment of the patient. This meeting allowed staff to contribute to decisions about treatment as well as ensuring the nursing staff were informed about safety for each child regarding mobility. The meeting also allowed for discharge planning to ensure that every child was safe to be discharged and was able to physically manage at home. A weekly psychosocial meeting was also developed and so the psychological and social factors for each child and their family could also be considered and supported.

**Case report - Key learning points:**

The hospital planned and prepared for the Pandemic and staff were placed together to work in different ways. Because of the diversity of the skills of the staff it was possible to recognise the many systems that were affected by the disease and to pull together the expertise of the staff to be able to provide a high level and holistic clinical management for each and every child. It has also been possible to explore outcome measures and to be able to work with each other and to learn and discuss treatments moving forwards.

The speed in which a completely new service was established was impressive especially as there had been a misunderstanding initially that children would probably not be severely affected by COVID-19.

The importance of physiotherapy treatment in order to enable these young people to regain strength, mobility and function was apparent and with the longer term follow up it is demonstrating that several of these patients need longer term care and treatment after discharge.

The outcome measures that are being used for the longer term follow up; CMAS, MMT8, 6 min walking test as well as neurological examinations and questionnaires to assess function and psychological well-being and fatigue are able to be used if other patients develop this disease and these measures can be used nationwide in order to enable a cohesive approach to managing PIMS-TR COVID-19.

